# The CANOVA Study Real-World Evidence of Biologic Treatments in Moderate-Severe Psoriasis in Italy: A Gender Perspective

**DOI:** 10.1089/whr.2021.0124

**Published:** 2022-05-02

**Authors:** Delia Colombo, Luca Bianchi, Gabriella Fabbrocini, Salvatore Corrao, Annamaria Offidani, Luca Stingeni, Antonio Costanzo, Giovanni Pellacani, Ketty Peris, Federico Bardazzi, Giuseppe Argenziano, Silvana Ruffolo, Paolo Dapavo, Carlo Carrera, Maria Concetta Fargnoli, Aurora Parodi, Marco Romanelli, Piergiorgio Malagoli, Alessandro Zullo, Fabio Ferri, Martina Fiocchi, Emanuela Zagni

**Affiliations:** ^1^ Novartis Farma S.p.A, Italy.; ^2^ Pharmacologist and Dermatologist Private Office, Milan, Italy.; ^3^ Policlinico Tor Vergata, Rome, Italy.; ^4^ A.O.U. Federico II, Naples, Italy.; ^5^ ARNAS Civico, Palermo, Italy.; ^6^ A.O.U. Riuniti Umberto I Lancisi Salesi, Ancona, Italy.; ^7^ Dermatology Section, Department of Medicine, University of Perugia, Perugia, Italy.; ^8^ IRCCS Istituto Clinico Humanitas, Rozzano, Italy.; ^9^ A.O.U. Policlinico, Modena, Italy.; ^10^ Fondazione Policlinico Universitario A. Gemelli-IRCCS e Università Cattolica, Rome, Italy.; ^11^ A.O.U. Policlinico S.Orsola-Malpighi, Bologna, Italy.; ^12^ Dermatology Unit, University of Campania, Naples, Italy.; ^13^ A.O. Cosenza Ospedale SS Annunziata, Cosenza, Italy.; ^14^ A.O.U. Città della Salute e della Scienza PO Molinette, Turin, Italy.; ^15^ Fondazione IRCCS Ca'Granda Ospedale Maggiore Policlinico, Milan, Italy.; ^16^ Dermatology, Department of Biotechnological and Applied Clinical Sciences, University of L'Aquila, L'Aquila, Italy.; ^17^ Clinica Dermatologica DiSSal Università di Genova/Ospedale-Policlinico San Martino IRCCS, Genova, Italy.; ^18^ A.O.U. Pisana Ospedale Santa Chiara, Pisa, Italy.; ^19^ Policlinico San Donato, San Donato M.se, Italy.; ^20^ MediNeos Observational Research, Modena, Italy.

**Keywords:** biologics, efficacy, gender differences, psoriasis, QoL

## Abstract

**Background:**

In psoriasis, several studies have indicated sex differences in clinical characteristics, type of treatment, and outcomes. A higher impact of psoriasis on quality of life (QoL) and a lower treatment satisfaction have been reported in women by different authors.

**Objectives:**

This article reports the results of a *post hoc* gender analysis of CANOVA study, aimed at assessing 16/24/52-week effectiveness of biologics in patients with moderate-severe plaque psoriasis.

**Materials and Methods:**

CANOVA was a real-world, multicenter, noninterventional, retro-prospective study conducted in 17 Italian hospital dermatology clinics.

**Results:**

Of the 669 eligible patients, 63.8% were men. Demographic and baseline characteristics and duration of disease were rather homogeneous between sexes. Slightly more women had been treated with biologics (50.4% vs. 46.5%) and had received ≥2 biologic treatment lines (17.2% vs. 12.4%) before study treatment. The most frequently used biologics were secukinumab, ustekinumab, adalimumab, and ixekizumab in both sexes. At 6 months, Psoriasis Area Severity Index (PASI) 75/90/100 responders were 90.8%/72.3%/45.3% of men and 89.2%/76.6%/48.2% of women. Sustained PASI responders were 79.5% of men and 75.9% of women. Treatment satisfaction was significantly lower in women at enrolment for all subscales, and was still lower at 6 months, no longer significantly. Gender distribution in Dermatology Life Quality Index total score classes showed a significantly greater effect of psoriasis on QoL in women, both at enrolment and at the 6-month follow-up.

**Conclusions:**

In conclusion, this gender analysis confirms in both genders the efficacy of biologics in psoriasis. However, women reported a greater impact of the disease on QoL and lower treatment satisfaction.

## What Is Already Known About This Topic?

- Considering the impact of sexual variables on diseases and treatments may potentially improve patients' care.
- In psoriasis, several studies have indicated sex differences in clinical characteristics, type of treatment, and outcomes.

## What Does This Study Add?

- No clinically relevant gender differences were observed in response to biologics in this real-life setting.
- For the same level of disease severity, women complained a greater impact of psoriasis on QoL and lower treatment satisfaction.
- These results reinforce the hypothesis that optimization of therapy may require a focus on gender.

## Introduction

Considering the impact of sexual variables on diseases can lead to improvements in care, especially in drug therapy, since gender differences in drug pharmacokinetics and tolerability have been identified.^1–3^ In psoriasis, several studies have indicated sex differences in various aspects of disease, including clinical characteristics, type of treatment, and outcomes.^4–6^ In particular, a higher impact of psoriasis on quality of life (QoL) and a lower treatment satisfaction have been reported in women.^7–12^

CANOVA (EffeCtiveness of biologic treAtmeNts for plaque psOriasis in Italy: an obserVAtional longitudinal study of real-life clinical practice) is a multicenter, observational, retro-prospective study, conducted in Italian dermatology clinics in adult patients with moderate-severe plaque psoriasis treated with biologics.^13^ The study's primary objective was to describe the clinical response rates after 16/24/52 weeks from biologic treatment line start. Secondary objectives included the description of treatment response by gender and the analysis of possible differences between sexes in patients' treatment satisfaction and QoL.

In this study, we report the results of gender analysis of the CANOVA study.

## Materials and Methods

### Study design and participants

CANOVA was an observational real-world study, involving both retrospective and prospective data, conducted in 17 Italian hospital dermatology clinics. Patients of both sexes ≥18 years of age, with moderate-severe plaque psoriasis, providing written informed consent before data collection, and treated with ≥1 biologics for psoriasis initiated between 24 weeks and 24 months before enrolment visit were enrolled. Patients who had interrupted the treatment before enrolment were also included. All treatments were administered according to current clinical practice. One enrolment visit, and one follow-up visit 6 months later (±1 month) were scheduled.

Assessment time points for the primary objective were 16 and 24 weeks, and 1 year after treatment initiation, so related data could have been collected retrospectively if these time points had occurred before the enrolment visit. Retrospective data were collected back to 24 months before enrolment, while the prospective observational period was framed between the enrolment visit and the 6-month follow-up visit (or until patient's withdrawal). Secondary objectives regarding the description of patients' treatment satisfaction and QoL from a gender perspective were evaluated at the enrolment and 6-month visits.

### Study objectives

The primary objective was to assess the proportion of patients achieving response to the study biologic treatment at 16 and 24 weeks and 1 year after treatment initiation. Secondary objectives included the evaluation of treatment response, patients' treatment satisfaction, and QoL by sex.

### Outcome measures

Clinical response to biologicals was defined as (1) achievement of Psoriasis Area Severity Index (PASI) 75 response at 16 and 24 weeks and 1 year and (2) a body surface area (BSA) ≤3% or a BSA improvement ≥75% at 16 weeks and afterward as a BSA ≤1% according to the National Psoriasis Foundation consensus conference.^14^ Sustained response at 1 year was defined as the achievement of (1) PASI 75 at week 16 and its maintenance at 1 year and (2) either a BSA ≤3% or BSA improvement ≥75% at week 16 and the maintenance of BSA ≤1% at 1 year.

PASI is the most extensively used psoriasis clinical severity score and is considered the reference scoring system against which other assessments tools are compared. It combines assessments of four body areas: head and neck, upper limbs, trunk, and lower limbs, and has shown high reproducibility and good inter-rater variability.^15^

A 75% improvement (PASI 75) is a common primary endpoint in clinical trials and is considered a valid measure for treatment decisions in real-world conditions.^16,17^ BSA has been suggested as a target goal by the National Psoriasis Foundation in a recent publication, which indicated a BSA ≤3% as acceptable response and a BSA ≤1% as target response after at least 3 months of a given treatment.^14^ It was demonstrated to be a highly reproducible method with moderate inter-rater variability. Study-appointed investigator of each center collected all available PASI and BSA assessments between treatment initiation and end of observation, in particular at the following time points: treatment initiation, 16 weeks, 24 weeks, and 1 year after treatment initiation.

The patients' satisfaction with the study biological treatment and the patient's QoL at the enrolment and follow-up visits were measured by means of the Treatment Satisfaction Questionnaire for Medication-9 (TSQM-9 subscale scores) and the Dermatology Life Quality Index (DLQI) questionnaire, respectively. The 9-item TSQM-9 provides scores on three scales—effectiveness, convenience, and global satisfaction—and was shown to be a reliable and valid tool, specifically in real-world studies. The three domain scores range from 0 to 100, with higher scores representing higher satisfaction.^18^ Adjusted mean scores were shown to range overall from around 66 among low treatment compliers to 84 among medium compliers.^19^

The DLQI is a 10-item questionnaire measuring the disability experienced by patients with different dermatologic conditions; its score range is 0–30, where higher scores represent a greater effect on QOL. It is widely used in psoriasis clinical trials and has demonstrated high sensitivity to change.^20,21^

### Statistical analysis

No formal statistical hypothesis was set. Mainly descriptive analyses were performed, both overall and stratified by gender. Moreover, confidence intervals (95% CIs) for the mean of numerical variables and for proportions of binomial variables (with the Clopper-Pearson exact method) were calculated. Distribution of patients according to DLQI total score and the mean TSQM-9 subscale scores were compared by gender at enrollment and 6-month follow-up by means of Fisher's exact test and through parametric Student's *t* test, respectively. Site monitoring, data management, and statistical analysis were performed by MediNeos (Modena, Italy). Database management and data analysis were performed using SAS Enterprise Guide v. 7.1 and SAS 9.4.

## Results

Between April 2018 and February 2019, 727 patients were enrolled and 669 (92.0%) were considered eligible after checking for inclusion/exclusion criteria. Of the eligible patients, 427 (63.8%) were males and 242 (36.2%) were females. The main demographic and clinical characteristics at enrolment are summarized in Table 1. The median observation period was 19.5 months (Q1–Q3 15.1–24.6). Fifty-seven (8.5%) of the eligible patients prematurely discontinued the study, mainly being lost to follow-up, somewhat more among males (9.8%, *N* = 42) than females (6.2%, *N* = 15).

**Table 1.** Demographic and Clinical Characteristics at Enrolment and Previous Biological Treatment Lines by Gender

	**Male patients (*N* = 427)**	**Female patients (*N* = 242)**
Age at enrolment (years)		
Mean (SD)	50.0 (13.2)	49.4 (15.9)
Hispanic/Caucasian		
*N* (%)	424 (99.3%)	241 (99.6%)
Baseline PASI		
Mean (SD)	14.3 (7.6)	13.6 (7.7)
N	342	188
No. of previous biologic treatments		
0	224 (53.5%)	118 (49.6%)
1	143 (34.1%)	79 (33.2%)
2	27 (6.4%)	18 (7.6%)
3	18 (4.3%)	15 (6.3%)
≥4	7 (1.7%)	8 (3.4%)
Unknown	8	4

All percentages are calculated on the number of patients with available data.

PASI, Psoriasis Area Severity Index; SD, standard deviation.

The overall mean duration of psoriasis since first diagnosis was 18.6 years (standard deviation [SD] 13.2; *N* = 588), whereas the overall mean duration of moderate-severe psoriasis was 15.8 years (SD 12.9; *N* = 457), with little difference between males (16.17 years; SD 12.45; *N* = 292) and females (15.07 years; SD 13.67; *N* = 165). Most patients (52.1%; *N* = 342) were receiving the study treatment as the first biologic for psoriasis (*i.e.*, naïve patients), 53.5% (*N* = 224) of males and 49.6% of females (*N* = 118). Females had received more previous biological treatment lines than males (Table 1).

The most used study biologic drug was secukinumab (*n* = 274; 41.0%), followed by ustekinumab (*n* = 169; 25.3%), adalimumab originator or biosimilars (*n* = 93; 13.9%), and ixekizumab (*n* = 81; 12.1%), and percentages were similar in both sexes. The complete list of biologics in use at enrolment by gender is reported in Table 2. Median (Q1–Q3) treatment duration during the study period was 17.6 (13.6–22.8) months in females and 18.1 (14.0–23.4) months in males.

**Table 2.** Type of Biological Treatments by Gender

	**Male patients (*N* = 427)**	**Female patients (*N* = 242)**
Biologic treatment		
Secukinumab	180 (42.2%)	94 (38.8%)
Ustekinumab	111 (26.0%)	58 (24.0%)
Adalimumab	57 (13.3%)	36 (14.8%)
Originator	53 (12.4%)	34 (14.0%)
Biosimilars	4 (0.9%)	2 (0.8%)
Ixekizumab	50 (11.7%)	31 (12.8%)
Etanercept	19 (4.5%)	11 (4.6%)
Originator	11 (2.6%)	6 (2.5%)
Biosimilars	8 (1.9%)	5 (2.1%)
Certolizumab	8 (1.9%)	11 (4.5%)
Golimumab	2 (0.5%)	1 (0.4%)

Topical treatments for psoriasis were used by 35.6% (*n* = 152) of males and 34.3% (*n* = 83) of females, mainly corticosteroids and vitamin D3 analogs for both genders (Table 3). Systemic pharmacological treatments for psoriasis other than biologics were received by 12.0% of females (*n* = 29) and 5.6% of males (*n* = 24), mainly methotrexate (4.1%, *n* = 10 and 2.6%, *n* = 11, respectively; Table 3).

**Table 3.** Concomitant Topical and Systemic Therapies for Psoriasis by Gender

	**Male patients (*N* = 427)**	**Female patients (*N* = 242)**
Patients receiving topical therapies during the observation period	152 (35.6%)	83 (34.3%)
Topical corticosteroids	73 (17.1%)	39 (16.1%)
Vitamin D3 analogs	60 (14.1%)	20 (8.3%)
Betamethasone/Calcipotriol^a^	58 (13.6%)	32 (13.2%)
Vitamin D Analog/Topical Steroid^a^	14 (3.3%)	8 (3.3%)
Urea	12 (2.8%)	8 (3.3%)
Salicylic acid	6 (1.4%)	5 (2.1%)
Topical retinoids	1 (0.2%)	1 (0.4%)
Other	51 (11.9%)	27 (11.2%)
Patients receiving systemic therapies for psoriasis other than biologics during the observation period	24 (5.6%)	29 (12.0%)
Methotrexate	11 (2.6%)	10 (4.1%)
Systemic corticosteroids	4 (0.9%)	6 (2.5%)
Oral small molecule (Apremilast)	5 (1.2%)	3 (1.2%)
Cyclosporin	3 (0.7%)	3 (1.2%)
NSAID	—	4 (1.7%)
Systemic retinoids	1 (0.2%)	3 (1.2%)
Fumarate (tablets)	1 (0.2%)	—
Other	5 (1.2%)	3 (1.2%)

^a^ Not included in the class “Topical corticosteroids” or “Vitamin D3 analogs.”

Response rates to the study treatments, defined as patients achieving PASI 75, together with PASI 90 and 100 responder rates at the study time points, stratified by gender, are detailed in Table 4.

**Table 4.** Psoriasis Area Severity Index Response to Biologic Treatments After 16, 24, and 52 Weeks Stratified by Gender

	**Male patients**		**Female patients**	
	***n*/*N***	**% (95% confidence interval)**	***n*/*N***	**% (95% confidence interval)**
Week 16^a^				
Patients achieving PASI 75	223/258	86.4 (81.6–90.4)	125/147	85.0 (78.2–90.4)
Patients achieving PASI 90	145/248	58.5 (52.1–64.7)	84/143	58.7 (50.2–66.9)
Patients achieving PASI 100	94/267	35.2 (29.5–41.3)	58/154	37.7 (30.0–45.8)
Week 24^a^				
Patients achieving PASI 75	227/250	90.8 (86.5–94.1)	116/130	89.2 (82.6–94.0)
Patients achieving PASI 90	172/238	72.3 (66.1–77.9)	95/124	76.6 (68.2–83.7)
Patients achieving PASI 100	116/256	45.3 (39.1–51.6)	66/137	48.2 (39.6–56.9)
Week 52^a^				
Patients achieving PASI 75	325/355	91.6 (88.2–94.2)	179/196	91.3 (86.5–94.9)
Patients achieving PASI 90	262/347	75.5 (70.6–79.9)	144/193	74.6 (67.9–80.6)
Patients achieving PASI 100	190/361	52.6 (47.3–57.9)	108/203	53.2 (46.1–60.2)

^a^ Week 16 = between weeks 10 and 20; week 24 = between weeks 20 and 30; week 52: between weeks 40 and 64.

BSA was assessed in a lower number of patients. BSA responders were 90.7% (95% CI 81.7–96.2; *n*/*N* 68/75) among females and 87.7% (80.5–93.0; 107/122) among males, at 16 weeks; 83.1% (73.3–90.5; 69/83) among females and 81.3% (74.3–87.1; 126/155) among males, at 24 weeks; and 79.9% (72.5–86.0; 119/149) among females and 83.7% (78.6–87.9; 220/263) among males, at 1 year. Sustained PASI responders were 75.9% (95% CI 68.0–82.7; *n*/*N* 107/141) among females and 79.5% (74.0–84.4; 198/249) among males. Sustained BSA responders were 51.4% (39.4–63.2; 38/74) among females and 57.7% (48.5–66.6; 71/123) among males.

The overall 43 patients (6.4%) who had switched from one biologic treatment to another during the study period were 27 males (6.3%) and 16 females (6.6%).

**FIG. 1.**
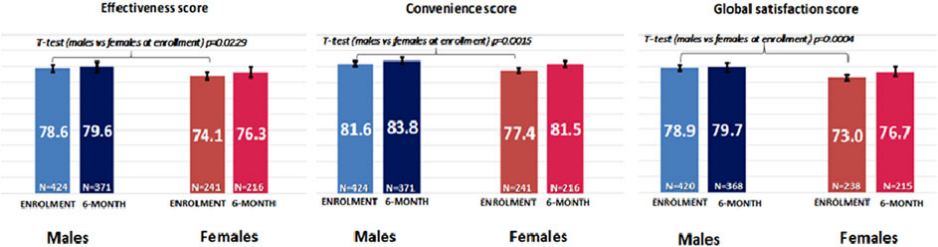
TSQM-9 scores at each visit stratified by gender. Score range: 0–100 points. Higher scores represent a higher grade of satisfaction. The patients with follow-up visit performed outside the tolerance window defined by Study Protocol (6 months ±1 month) were not excluded. Bars represent the mean TSQM-9 score values (reported in the center of bars) and whiskers indicate the 95% confidence interval limits for observed means; the number of patients considered is reported at the bottom part of the graph . *T*-test *p* values Effectiveness, Convenience, Global Satisfaction scores, males versus females at 6 months >0.05. TSQM-9, Treatment Satisfaction Questionnaire for Medication-9.

The overall mean scores for the three TSQM-9 subscales—effectiveness, convenience, and global satisfaction—were 76.9 (SD 24.3; *N* = 665), 80.1 (16.5; *N* = 665), and 76.7 (20.6; *N* = 658), respectively, at enrolment visit, and slightly increased at the 6-month follow-up visit to 78.4 (23.8; *N* = 587), 83.0 (15.6; *N* = 587), and 78.6 (20.4; *N* = 583), respectively. Treatment satisfaction with the study biological medication, measured by TSQM-9, was significantly lower in women at enrolment for all subscales, that is, effectiveness (*T*-test [males vs. females] *p* = 0.0229), convenience (*p* = 0.0015), and global satisfaction (*p* = 0.0004). At the 6-month evaluation, all three TSQM-9 subscales were still slightly lower in women, but no longer significantly (Fig. 1).

The overall mean DLQI total score was low at enrolment and decreased between the enrolment and 6-month visits, from 2.3 (SD 3.9; *N* = 666) to 1.8 points (SD 3.6; *N* = 588). The distribution of males and females in the DLQI total score classes shows a statistically significant difference both at enrolment (Fisher's exact test *p* = 0.0008) and the 6-month follow-up (*p* = 0.0087; Table 5), with women suffering a greater impact than men on their life. There was an overall high proportion of patients (64.4% at enrolment and 70.4% at 6-month follow-up) reporting to have no effect at all on their QoL (*i.e.*, DLQI total score 0–1 points), with a specularly higher proportion in males compared to females (Table 5).

**Table 5.** Patients' Quality of Life (Dermatology Life Quality Index) at Each Visit Stratified by Gender

	**Male patients**	**Female patients**	***p*** ^**a**^
At enrolment	*N* = 426	*N* = 240	
DLQI total score (classes)			
0–1 (no effect at all on patient's life)	295 (69.2%)	134 (55.8%)	0.0008
2–5 (small effect on patient's life)	93 (21.8%)	60 (25.0%)	
6–10 (moderate effect on patient's life)	21 (4.9%)	29 (12.1%)	
11–20 (large effect on patient's life)	14 (3.3%)	15 (6.3%)	
21–30 (extremely large effect on patient's life)	3 (0.7%)	2 (0.8%)	
At the 6-month follow-up visit	*N* = 372	*N* = 216	
DLQI total score (classes)			
0–1 (no effect at all on patient's life)	277 (74.5%)	137 (63.4%)	0.0087
2–5 (small effect on patient's life)	69 (18.5%)	46 (21.3%)	
6–10 (moderate effect on patient's life)	16 (4.3%)	17 (7.9%)	
11–20 (large effect on patient's life)	9 (2.4%)	14 (6.5%)	
21–30 (extremely large effect on patient's life)	1 (0.3%)	2 (0.9%)	

Higher score indicates higher impairment on quality of life. The patients with follow-up visit performed outside the tolerance window defined by Study Protocol (6 months ±1 month) were not excluded.

^a^ Fisher's exact test.

DLQI, Dermatology Life Quality Index.

## Discussion

The patients of this study were enrolled in different Italian hospital dermatology clinics according to wide and simple inclusion/exclusion criteria to reflect as much as possible the plaque psoriasis population observed in clinical practice. Patients were predominantly males (64%), consistent with the fact that only patients with moderate-severe psoriasis were enrolled, and men have been reported to have more severe psoriasis than women.^5,22–24^

Twice as many women as men were treated with systemic drugs other than biologics, in line with previous data, showing that women are more likely than men to be prescribed biologics and overall systemic therapy.^24^ Although there were no relevant differences in the duration of illness, more women than men had been previously treated with biologics (50% vs. 47%) and women had also received more previous biological treatment lines. This latter observation is also in line with the literature.^24^ We hypothesize that this may be related to the reported lower satisfaction with treatment among women with psoriasis,^6,10^ which is confirmed by our results, showing at enrolment significantly lower scores among women for all TSQM-9 domains.

Among the investigated biologics, secukinumab was the most used, followed by ustekinumab and TNF inhibitors (17.8%)—mainly adalimumab—a ranking that roughly reflects the prescription patterns for moderate-severe plaque psoriasis in Italy. Anyway, the range of currently available biologics for moderate-severe plaque psoriasis was rather completely represented in our study, with no relevant gender difference.

The main objective of this gender analysis was to assess possible differences by gender in treatment response, QoL, and treatment satisfaction.

Response to biologic treatments was overall high already at week 16 with no gender difference and no clinically relevant gender difference in total PASI 75/90/100 and BSA response rates at all study time points. Sustained responder rates were also overall high and similar between the two sexes, with males having a numerically slightly higher proportion of responders both for PASI and BSA. In the literature, we found only one study from the US Corrona registry addressing differences between genders in response to biologic treatments in psoriasis, which unlike our study reported that female sex was significantly associated with a decreased likelihood of achieving a response to biologic agents.^25^

However, despite having similar success rates than men, the women of our cohort reported a greater impact of psoriasis on the QoL of women than of men, for the same level of disease severity, consistently with published data reporting greater levels of psychological distress in women,^7,11,26^ at least partially explained by the impact of psoriasis on physical appearance, which usually matters more to women for whom the disease is also more difficult to hide.

Furthermore, the patient perspective analyzed from German and Swiss psoriasis registries showed that women complained greater feeling of depression, worse sleep quality, and less everyday productivity.^9^ Also, the PSYCHAE Italian multicenter study pointed out that the psychological distress was worse in women with psoriasis than in men, regardless of severity of psoriasis.^8^

Concomitant topical treatments were taken by about 35% of patients of both sexes. The proportion of patients taking systemic therapies, in addition to the biological, was double in women than in men, suggesting again that this might be related to women's lower treatment satisfaction rather than lower efficacy of biologics.

The global CANOVA study has limitations, mainly related to its observational, real-world nature, including the high heterogeneity of the patient population, the different durations of observation, and a certain inhomogeneity in the quality of collected data, depending on the mainly retrospective design of the study. A further limitation of this gender analysis is that the study was not specifically designed and sized for the gender comparison. On the other hand, this study has the advantage of providing data relating to typical patients accessing dermatology clinics in the Italian clinical practice.

## Conclusions

In conclusion, this gender analysis of the main results of the CANOVA study confirms in both genders the effectiveness of biologic agents observed in the overall population in dramatically reducing the cutaneous lesions of moderate-severe psoriasis. Women complained a greater impact of the disease on QoL and a lower treatment satisfaction. These differences in the burden of disease perceived by women compared to men suggest once more that optimization of therapy may require gender attention and confirm the need for studies evaluating the gender impact of diseases and treatments.

## Notes

**Cite this article as:** Colombo D, Bianchi L, Fabbrocini G, Corrao S, Offidani A, Stingeni L, Costanzo A, Pellacani G, Peris K, Bardazzi F, Argenziano G, Ruffolo S, Dapavo P, Carrera C, Fargnoli MC, Parodi A, Romanelli M, Malagoli P, Zullo A, Ferri F, Fiocchi M, Zagni E (2022) The CANOVA study real-world evidence of biologic treatments in moderate-severe psoriasis in Italy: a gender perspective, *Women's Health Report* 3:1, 450–457, DOI: 10.1089/whr.2021.0124.

## Abbreviations Used

BSA: body surface area
DLQI: Dermatology Life Quality Index
PASI: Psoriasis Area Severity Index
QoL: quality of life
TSQM-9: Treatment Satisfaction Questionnaire for Medication-9
